# A Review of Impact of Textile Research on Protective Face Masks

**DOI:** 10.3390/ma14081937

**Published:** 2021-04-13

**Authors:** Jiri Militky, Ondrej Novak, Dana Kremenakova, Jakub Wiener, Mohanapriya Venkataraman, Guocheng Zhu, Juming Yao, Arun Aneja

**Affiliations:** 1 Department of Material Engineering, Faculty of Textile, Technical University of Liberec, 46117 Liberec, Czech Republic; dana.kremenakova@tul.cz (D.K.); jakub.wiener@tul.cz (J.W.); 2 Department of Nonwovens and nanomaterials, Faculty of Textile, Technical University of Liberec, 46117 Liberec, Czech Republic; novak.ondra1@seznam.cz; 3 School of Materials Science and Engineering, Zhejiang Sci-Tech University, Hangzhou 310018, China; zgc100100@hotmail.com (G.Z.); yaoj@zstu.edu.cn (J.Y.); 4 Department of Engineering, East Carolina University, Greenville, NC 27858, USA; anejaap@gmail.com

**Keywords:** textiles, facemask, COVID-19, nanoparticles, anti-viral

## Abstract

COVID-19, classified as SARS-CoV-2, is causing an ongoing global pandemic. The pandemic has resulted in the loss of lives and has caused economic hardships. Most of the devices used to protect against the transmission of the novel COVID-19 disease are related to textile structures. Hence, the challenge for textile professionals is to design and develop suitable textile structures with multiple functionalities for capturing viruses, passivating them, and, at the same time, having no adverse effects on humans during the complete period of use. In addition to manufacturing efficient, biocompatible, and cost-effective protective face masks, it is also necessary to inform the public about the benefits and risks of protective face mask materials. The purpose of this article is to address the concerns of efficiency and efficacy of face masks by primarily reviewing the literature of research conducted at the Technical University of Liberec. The main focus is on the presentation of problems related to the specification of aims of face mask applications, mechanisms of capture, durability, and modes of sterilization. The recommendations, instead of conclusions, are addressed to the whole textile society because they should be leading players in the design, creation, and proper treatment of face masks due to their familiarity with the complex behavior of textile structures and targeted changes of structural hierarchy starting from polymeric chains (nano-level) and ending in planar textile structures (millimeter level) due to action by mechanical, physical and chemical fields. This becomes extremely critical to saving hundreds of thousands of lives from COVID-19.

## 1. Introduction

Two years ago, Reuters (Business News 15 March 2018, 11:59) published a detailed report entitled “Toyobo to pay $66 million in US bulletproof vests fraud case”, which described a long process that led to the conviction of a top Japanese company for introducing Zylon fiber in “bulletproof vests”. It was found that these vests did not work in real-life conditions, leading to several fatal injuries to police officers. It seemed almost unbelievable as Toyobo and the American partner who marketed these vests had performed extensive ballistic tests comprehensively and successfully. However, the important fact that was missed was that the tests of “fresh” material (from production) and tests of the same material after a certain aging period under normal conditions can be so different that a working material gradually becomes inefficient in its use. A similar perspective is applicable to face masks which can potentially impact humans more.

Compared to bulletproof vests, masks are extremely complex. Humidity, thermal conditions, and light intensity with the normal use of protective face masks, respirators, and other devices that protect against bacteria and viruses are generally more demanding than with the use of bulletproof vests. More complexities arise in the production of polymers, fibers, and fabrics intended for virus protection agents, several auxiliary chemicals commonly used, from catalysts, through processing aids, to substances of only aesthetic significance (matting, color pigments in the mass). Through continued exposure of the human body through breathing, these chemicals can potentially cause worse effects than the impact of the viruses themselves. Much of this article is based on the extensive research performed by the authors and the results of the research have been provided as supporting evidence for the recommendations made. These recommendations are extremely critical at this point because hundreds of thousands of lives may be saved, which otherwise will be lost, not just to the current pandemic caused by the COVID-19 virus, but also to similar calamities in the future.

Coronavirus disease (COVID-19) is a novel infectious disease caused by the virus family of severe acute respiratory syndrome (SARS) and classified as SARS-CoV-2; it is a positive-sense single-stranded RNA virus that is causing an ongoing global pandemic [1]. Most of the devices used to protect against the transmission of the novel COVID-19 disease are more or less related to textile structures. It becomes necessary that textile professionals design and develop suitable textile structures with multiple functionalities for capturing viruses, passivating them, and, at the same time, having no adverse effects on humans during the complete period of use. In addition to manufacturing efficient, biocompatible, and cost-effective protective face masks it is also necessary to create awareness about the benefits and concerns of protective face mask materials. The purpose of this article is not to cause concern, but to avoid a similar fate for a face mask to that suffered by the Zylon fiber. This means supplying and recommending only those materials that have passed actual tests under the conditions of use for the recommended time. Tests must focus on both the loss of efficiency and the possible simultaneous decline in product performance due to aging of materials through combined hydro-, thermo-, photo-degradation. The specific features of textile materials connected with their impact on construction, use, and sterilization of protective face masks are discussed. In this review, we utilize the research conducted at the Technical University of Liberec to explore problems related to the specification of aims of face mask application, mechanisms of capture, durability, and mode of sterilization. The recommendations instead of conclusions are addressed to the whole textile society because they should be leading players in the design, creation, and proper treatment of face masks during use. No other specialist is familiar with the complex behavior of textile structures and targeted changes of structural hierarchy starting from polymeric chains (nano-level) and ending at planar textile structures (millimeter level) due to the action of mechanical, physical, and chemical fields. This becomes extremely critical at this point because hundreds of thousands of lives may be saved, which otherwise will be lost, not just to the current pandemic caused by the COVID-19 virus, but also for similar future calamities.

## 2. Literature Review

### 2.1. Coronavirus Responsible for COVID 19

**Figure 1:**
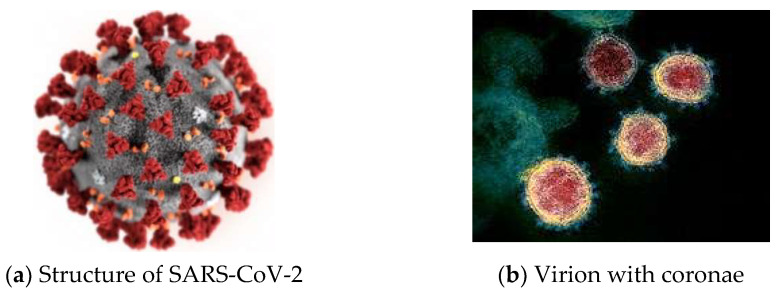
Coronavirus SARS-CoV-2 (size 50–200 nanometers) [1].

SARS-CoV-2 has four structural proteins, known as S (spike), E (envelope), M (membrane), and N (nucleocapsid) (see Figure 1a) [1]. The virion with coronae is shown in Figure 1b.

The N-protein holds the RNA genome, and the S, E, and M proteins, together, form the envelope of the virus body. The S-protein is responsible for allowing the virus to attach to the membrane of a host cell. Chemically, these are primarily glycoproteins containing oligosaccharides covalently attached to the side groups of the polypeptide chains. The entry mechanism of coronaviruses into human cells is a complex phenomenon and requires several proteolytic processes. The entry of coronaviruses is mediated by a trans-membrane S protein, which is present in the form of a homotrimer on the viral envelope. There is a diversity of protein domains, which interact with the variety of host receptors. Indeed, different species use different cellular receptors to get entry into the host cells. S proteins of SARS-CoV2 bind to the host receptor known as, angiotensin-converting enzyme 2 (ACE2) [2]. The surface stability of SARS-CoV-2 in aerosol form (such as when exhaled by infected persons) compared to SARS-CoV-1 was quantitatively investigated [3]. It was found that the virus can remain viable and infectious in aerosols for many hours and on surfaces (especially polymeric) for up to several days. The stability of SARS-COV-1 (source of SARS disease) and SARS-COV-2 virus in the air and on various surfaces were investigated. SARS-COV-2 was most stable on plastic and stainless steel and the viable virus was detected up to 72 h after application. On the other hand, after 4 h, no viable virus was found on a copper surface. Copper ions form bonds with glycoproteins (and probably strongly reduce coronavirus replication in human cells) [4].

### 2.2. Textile Materials

Textile materials and structures are used by humans from birth until death. They are composed of textile fibers mainly joined together by surface friction forces. Textile fibers (natural fibers/synthetic fibers) are a very specific form of polymer because their geometry and behavior are unique. Fiber is a generic name for long (length *l* approx. 10^−2^ to 10^−1^ m) thin (diameter *d* approx. 10^−6^ to 3. 10^−5^ m) rod-like formations prepared from polymeric or non-polymeric substances. The typical length to diameter ratio *l*/*d* is about 10^3^ [5]. Many important properties of fibers are not directly related to fiber chemical compositions but to supermolecular structure and morphology created during the growth phase in case of natural fibers, and after spinning, by operation of drawing and thermal treatment (setting) in case of chemical and synthetic fibers [5]. Polymeric fibers have a typical fibrous structure characterized by the hierarchy of long thin element bundles (molecular chains, micro-fibrils, macro-fibrils) oriented preferably in the fiber axis direction and having more or less ordered three-dimensional arrangements. (semi-crystalline state). Due to this special structure, fibers have high anisotropy of physical and mechanical properties. Textile fibers have special organoleptic properties (luster, hand), technological properties (length, strength, crimp, surface roughness, etc.), and utility properties (sorption, ability to set, abrasion resistance, etc.). Their structural features, properties, and behavior are described in detail, e.g., in [6]. For the construction of face masks, hydrophobic synthetic fibers are usually selected but natural fibers have the characteristic of attracting, e.g., water droplets on their surface.

The majority of users, laymen, may think that based on extensive use of textiles during their life, they understand textile behavior and the right applications. This is an incorrect assumption due to the new developments happening in the textile domain. Textile specialists and fiber manufacturers are routinely producing textiles that go beyond standard fiber materials and change their areas of application. Not without reason, in Japan, they decided to label fibers with new effects with the term “Shingosen” (new fiber) to suppress users’ experience with fibers of similar chemical compositions, but with significantly different behaviors. Many new effects on textiles are obtained by finishing with chemical agents. When the fabric treatment proves to be sensitive at elevated temperatures, it is simply recommended to lower the washing temperature. Instead of washing, the textiles are then only soaked in tepid water, which becomes beneficial to many microorganisms. Temperature is replaced by detergents, which are often not known to affect treated fabrics. The textile fabrics themselves have some properties that directly impact their use for protective face masks. They generally have poor permeability to UV radiation, especially of shorter wavelengths. This limits the use of sterilization by UV C radiation (with shorter wavelengths below 280 nm) only for textile surfaces, which is often not enough.

For cotton fabrics, the amount of penetrating UV is usually small. Thus, all methods of virus mitigation and antimicrobial treatments based on photocatalytic oxidation (e.g., with TiO_2_) are non-functional if the photoactive layer is covered with, e.g., cotton fabric. The use of highly effective antimicrobial agents (e.g., Triclosan) have already been banned and withdrawn from the market. Perfluorinated compounds containing perfluorooctanoic acid and their salts have been included by the Stockholm Convention in the list of prohibited substances and their use is restricted by European Directive 2006/122/EC (this applies, for example, to Gore-Tex material). It may not be placed on the market or used as a substance or constituent of preparations in a concentration equal to or greater than 0.005% by mass. Under REACH directives, the use of substances containing perfuloroalkanes will be completely banned by 2020. Titanium dioxide is likely to face a similar fate. For each kind of face masks finishing, it will therefore be necessary, not only to verify their real functionality, but also the possible health risks.

At present, modern textile structures with possible antimicrobial treatments [7], including simple and effective sterilization, are an essential part of protective face masks where possible re-use is planned. This brings together several yet-unsolved problems [8,9] that, as a result, can make the impact of the use of these face masks on the health of wearers worse than being infected by viruses [6,10,11,12]. It should be kept in mind that the total wearing time of protective face masks associated with the possible release of unhealthy particles can be extremely long. In accumulation, the concentration of inhaled particles in the human body may exceed permissible limits, thus having a negative effect.

### 2.3. Protective Face Masks

The basic problems related to the construction and usability of virus protection textiles include specification of purpose of use, mechanisms of capture, durability, and mode of sterilization.

#### 2.3.1. Specification of the Purpose of Use of Face Masks

It is important to differentiate the issues of face masks: one for preventing viruses from entering the mouth and nose in healthy people (“*I protect myself*” face masks), i.e., for prevention—RP, and the second being face masks preventing the spread of viruses from the mouth and nose in infected people (“*I protect you*” face masks), i.e., for the protection of the environment—RO.

In RP face masks, inhalation is critical, when the mask is pressed against the face and the virus penetrates from the outside. The intensity of the virus’s attack, mainly in water droplets, is usually only moderate or low and for short time only. The main aim here is the attachment of water droplets on the surface of the mask and avoiding penetration to the nose or mouth.

In the case of RO face masks, exhalation is critical, when due to local build-up of pressure they can be separated from the face and contaminated moist air penetrates in places with the lowest air resistance. Hence, the primary aim is avoiding the spread of droplets containing viruses or mitigating their impact quickly. This is still not easy because mitigation of viruses by the majority of techniques is relatively slow—more than ten minutes. Most efficient techniques are dangerous for human cells as well. For RO face masks, it will be probably necessary to add another special intermediate removable layer with effective antiviral functions [13].

**Figure 2:**
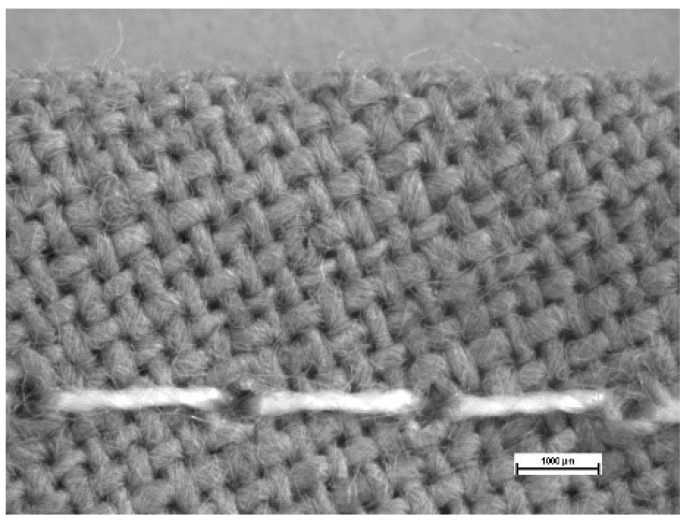
Holes in face mask created by a sewing needle (magnification: 1000 µm).

The local pressure conditions under face masks can be changed by their construction and incorporation of seams. A sewing needle creates relatively large holes (see Figure 2). Thus, area nearby seams are therefore the weakest point fastening the process of moist air penetration.

Types of seams that are suitable for cases where it is necessary to reduce the penetration of air are known. However, it is not widely used in face masks. The problem is how to determine which kind of face masks people with a possible viral infection should use. For safety reasons, it will be necessary to require the use of RO masks for these individuals. In the construction of RO face masks, it should be investigated how and where air flows through a multilayer system with loosely stacked layers (textile and intermediate antiviral removable layer) with varying air resistance, and where are critical locations for virus penetration.

#### 2.3.2. Capturing Mechanisms

**Figure 3:**
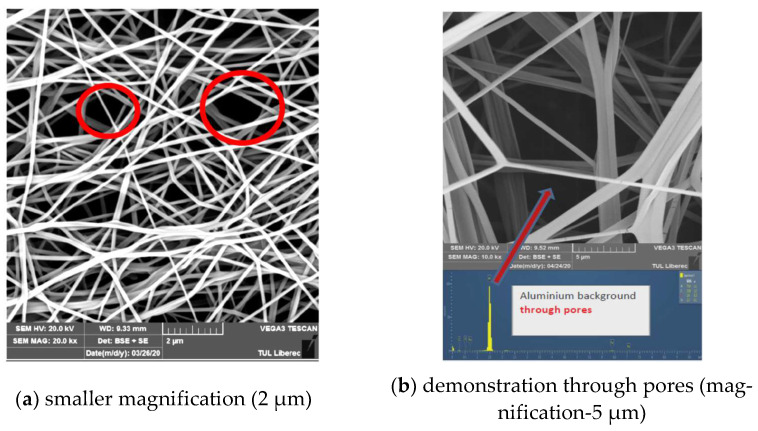
Pores in melt-blown PP (polypropylene) nanolayer used for face masks.

Principally there are different mechanisms for capturing or deactivating viruses. The majority of them are dangerous for human cells as well. For some mechanisms, the kinetics is too slow to be used for the real functioning of face masks. Other mechanisms are too dangerous for humans and can be used for external sterilization only. Many face masks work on the principle of mechanical capture. A thin barrier layer capable of stopping the spread of the virus (for electrospun “nanofiber” layers it is approximately 2 micrometers high) should ideally have pores smaller than the size of the “dry” virus (from 50 nm). Due to limited thickness, the bigger portion of pores is straight through-pores (see Figure 3). It is demonstrated in Figure 3b that the pores shown through the background are composed of only alumina.

A thicker barrier layer capture system is based on the increase of tortuosity and larger no throughput pores.

The term “nano” suggests that it is a material with pores in the nano range. In many cases, this term is used as a desirable attribute that is enjoyed by the general public. Experts understand according to the current European standard (nanotechnologies—terminology and definitions for nano-objects—nanoparticle, nanofibre, and nanoplate: ISO/TS 80004-2: 2015 E), nanomaterials are defined as materials that have at least one dimension (here diameter) in the range 1–100 nm. This range of diameters of the fiber segments in “nano” face masks or “nanolayers” prepared by electrospinning usually exceeds this limit (see Figure 3). These materials are then not “nano” according to the standard. The required pore size cannot be achieved using standard “nanofiber” layers, where there is a wide pore size distribution from nano to micrometers. By layering the individual “nanofiber” layers, it is possible to create a system with a much narrower pore size distribution [14], but the problem is how to combine these layers? A serious limitation is that the “nano-fibrous” layers are mechanically unstable and can, in a short time, cease to protect due to mechanical macro-breakage of the structure (formation of holes) and thus directly endanger the wearer. The problem is also caused by the fiber segments, which are released after only several cycles of abrasion (friction between the layers or mechanical action from the outside). It is, therefore, necessary to verify the durability of face masks containing “nano-fibrous” structures during use to test the released fragments ([14]) like the so-called microplastics.

For mechanical barrier layers of higher thickness, the tortuosity of the pores will play an important role, as viruses penetrating the water droplets can be deposited on the pore walls. Table 1 shows the differences between types of electrospun polyamide (PA) 6 nano-membrane and spunbond polyamide (PA) 6 micro-membrane [15].

The typical porosity of micro membranes is much higher than that of nanomembranes and the mean path from one side to another is relatively very long. Droplets will therefore be more frequently in contact with fiber surfaces with the possibility of attachments. We hypothesize that coronaviruses glycoproteins can adhere to sites with polar groups (as is e.g., surface of cotton or PA 6) capable attract them by electrostatic hydrogen bonds. When viruses spread from infected people, they are probably mostly in water droplets of a much larger size. The overall mean cough droplet size distribution was found to be 0.62–15.9 microns [16]. These droplets can easily change shape (like all liquids) and penetrate through significantly smaller pores than solid particles. The assessment of the quality of the capture by the penetration of solid particles or oil particles used, e.g., for testing of filtration materials is therefore only very indicative.

One of the major reasons used to promote the use of nanofibers assembly is that they have extraordinary relative surface area *S_r_* [m^2^/g] defined as surface area per mass. For fibrous assembly with fiber diameter *r* [µm] and fiber density $\rho_{F}$ [kg/m^3^] it is

(1) $$S_{r}=\frac{2000}{r\;\rho_{F}}$$

Relative surface area is inversely proportional to fiber diameter. A major problem in the fibrous layer is due to the limited range of thickness *h* [mm]. Nano layers with planar mass *gsm* [g/m^2^] about 1 g/m^2^ are very thin. Planar mass *gsm* is related to volume porosity of fibrous layer *P*_o_ and the thickness of the layer

(2) $$gsm=\left(1-Po\right)\;h\;\rho_{F}$$

Fibrous layers macro-surface area *S_m_* [m^2^] is a product of the width and length. It is possible to calculate relative macro-surface area *S_mr_* = 1/*gsm* [m^2^/g] per gram as well. Quantity *S*_mr_ is, for thin layers, very large. In reality, the use of textile layers is based on macro surface area *S_m_* and not on weight because textile layers cover some other areas, e.g., the human body or in filters. It is the reason for use quantity *S_sr_* [m^2^/m^2^], i.e., relative surface area of fibrous layers per macro surface area *S_m_.* After some calculation, it is possible to obtain relations

(3) $$S_{sr}=1000\;\frac{2\;(1-P_{o})\;h}{r}=S_{r}\;gsm$$

where *P_o_* is the total volume porosity of fibrous assembly defined as one minus fiber packing density in the fibrous layer. For the case of nanofibrous and microfibrous layers, examples are calculated and relative surface areas are given in Table 1. It can be seen that there are quite different results and *S_sr_* for the microfibrous layer is much higher than for the nanofibrous layer. Therefore, nanofibrous layers have, in reality (the same macro surface *S_m_*), a much smaller surface area than the microfibrous layer.

#### 2.3.3. Filtration Performance Control

The use of layers composed of submicron fibrous segments is motivated by the effectivity of mechanical virus capture. A serious limitation is a small thickness (through pores) and the negative influence of breathing under the mask. One possibility for avoiding these limitations is to use modified melt blow technology for the preparation of thicker layers containing a random mixture of both micro and nanofibers [17]. Standard melt blowing is a production process providing fibers with a diameter of micrometers or tens of micrometers. Common technology leads to fiber diameters from approx. 2 micrometers up to 20 micrometers. The mechanical properties of the melt-blown layers are relatively low, while the filtration properties are excellent. Modified melt blowing can also produce parts of fibers with a diameter under 1 micrometer [17]. The melt blowing applied for the production of layers used here created a mixture of fibers with different diameters in one layer. Advantageously, compared to other manufacturing technologies using electrostatic forces to produce fibers (such as electrospinning), melt blowing has a very low residual electric charge.

##### Layers Preparation

Seven different layers with different planar densities and porosity were produced by the modification of conditions (rate of production mainly) of the melt blowing process [17]. Polypropylene granules (Exxon-mobile) were used for the experiments. By varying the process parameters (rate of winding), layers with different planar densities were prepared (see Table 2).

**Figure 4:**
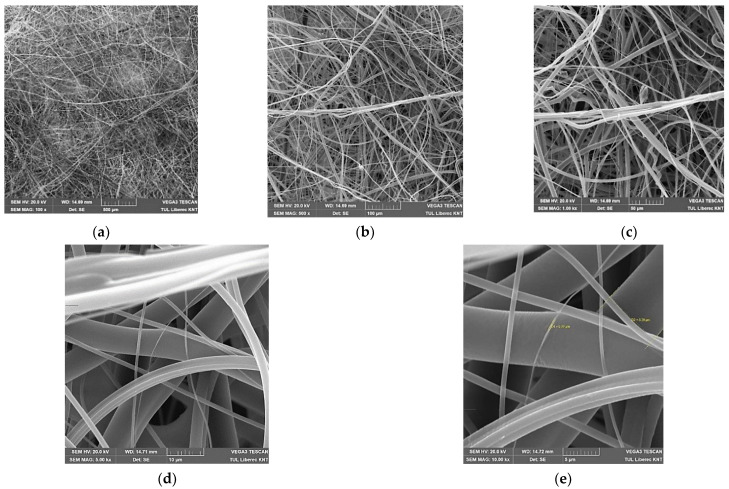
SEM images of prepared melt-blown layers at different magnifications: (**a**) 500 µm, (**b**) 100 µm, (**c**) 50 µm, (**d**) 10 µm, (**e**) 5 µm.

In Figure 4 typical fibrous layer structure images at different magnification are shown.

##### Filtration Efficiency

Filtration properties were measured on a PALAS 1000 MSP device. Parameters of testing are shown in Table 3. The measurements were realized in accordance with standard ISO 16890. ISO 16890 refers to particulate air filter elements for general ventilation having an ePM1 efficiency less than or equal to 99% when tested.

Filtration efficiency characteristics are given in Table 4.

**Figure 5:**
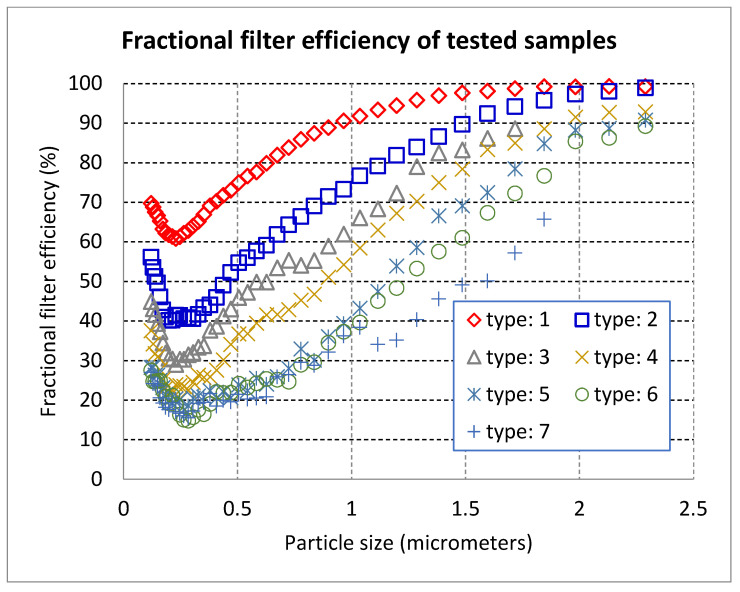
Fractional filter efficiency of samples.

Results of testing are summarized in Table 5 and Figure 5.

For a better understanding of results, the values of filtration characteristics required for different filtration purposes are shown in Table 5.

From Table 5 and Table 6, is seen that type 1 and 2 layers are suitable for filtering viruses, nanoparticles, and exhaust gasses; layer 3 is suitable for filtration of bacteria, fungi, mold spores, and pollen (ISO e_PM2.5_), and other layer types are classified as ISO e_PM10_. The first two materials are very suitable for the production of high-efficiency breathing masks and respirators due to the high filtration efficiency and low-pressure drop. The maximal efficiency for particles with a diameter of 2.23 μm was 99.25%. The main advantage of the modified production technique is very high productivity compared to electrospinning and thus a very low price for the product. By changing of parameters of the modified melt-blowing process it is possible to tune filtration efficiency and breathing comfort simultaneously [18,19].

#### 2.3.4. Antimicrobial Mechanisms

A review of typical antiviral agents for mitigation of the impact of viruses has been already published [10]. In our recent book [6], major types of antiviral mechanisms are summarized. Some of them are known for destroying organic materials and some of them are selectively active. One practically universal option is the destruction of all types of microorganisms, including viruses, via local photo-oxidation. For this purpose, titanium dioxide (TiO_2_ (titanium)) has traditionally been used to form pairs of free electrons and holes in the conductive and valence bands when activated by light, particularly in the UV region (wavelength < 390 nm). These free electrons and holes react with oxygen and hydroxyl groups from atmospheric moisture to form superoxides (·O_2_^−^) and hydroxyl radicals (·OH), destroying organic pollutants, bacteria, and viruses [20]. The main limitation is the requirement to have illumination by a source of UV light and the slow rate of destruction of organic material.

**Figure 6:**
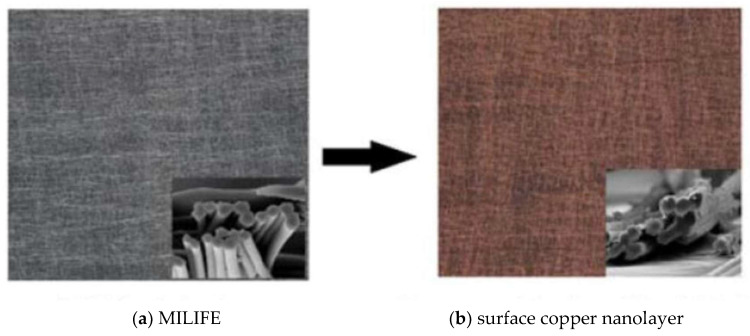
Composite nonwoven MILIFE with surface deposition of copper.

Possibly, the effective action of copper is due to the formation of Cu^+^ and/or Cu^2+^ ions. On the other hand, in publication [21] they found that Zn^2+^ is a good viral inhibitor for coronaviruses. One promising material for viral inhibition is a copper particle-based nanolayer on special composite nonwoven structures (MILIFE^®^). This nonwoven structure is a product of JX Nippon ANCI Corporation, and is composed of a machine-directional (MD-oriented) and cross-directional (CD-oriented) layer of PET monofilaments. Both layers are thermally bonded together (see Figure 6a). MILIFE is a promising material for surface metals deposition because it is composed of a dense network of monofilaments connected by fixed points [22,23]. Plasma pretreatment with autocatalytic activation can be used for copper deposition (see Figure 6b) from a strong alkaline bath containing copper salts and a reduction agent (borohydride). The copper surface concentration is about 3 g m^−2^ [23].

It is also possible to use special dyes containing copper complexes (phthalocyamines, metal-complex dyes).

**Figure 7:**
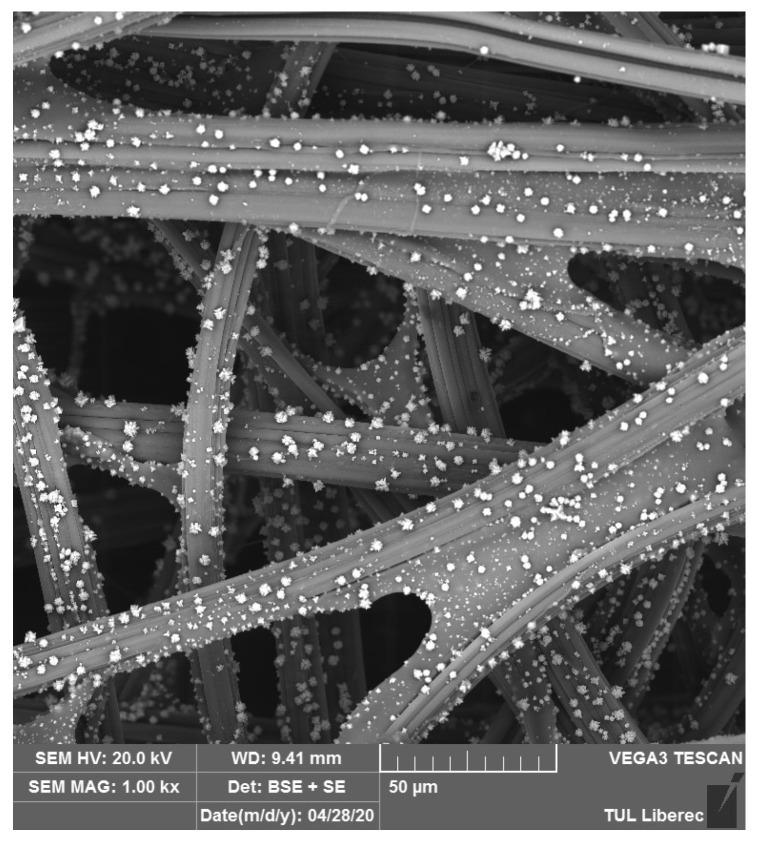
Deposition of Cu_2_O on the surface of viscose fabric.

One promising way to prepare an antiviral layer is to use deposition of Cu_2_O on viscose (see Figure 7) or polyester fabric. In our study, Cu_2_O was deposited on textile materials in an aqueous solution of Cu^2+^ and sodium citrate complex alkalized with Na_2_CO_3_ at elevated temperatures 50–60 °C in a water bath. At lower temperatures, the reaction is much slower, but may result in a better surface distribution and smaller particles of precipitate with a better adhesion to the fiber surface. Surface pre-treatment or activation of the polymer material prior to the reaction may improve the performance of Cu_2_O deposition. Based on preliminary tests, this material shows very effective antiviral behavior.

Generally, all nanoparticles have gained increasing attention due to their potential antimicrobial effects. There are three main mechanisms by which nanoparticles can act as antiviral agents. First, nanoparticles can inhibit the virus directly by penetrating the viral coronae. Different nanoparticles are effective for enveloped and some for nonenveloped viruses. Second, nanoparticles can bind to viruses preventing them from attaching to host cells when entering the body. Third, nanoparticles can prevent replication of a virus [24]. For surface finishing purposes, the first two mechanisms are important as the virus can be inactivated on contact with an antiviral surface or fiber.

**Figure 8:**
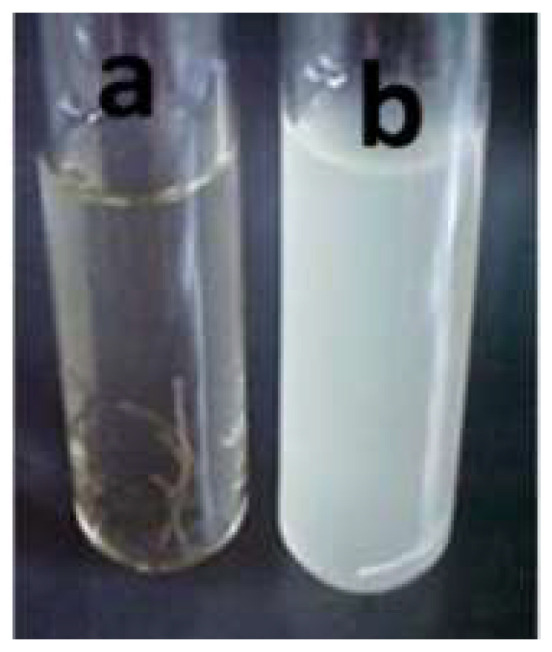
Difference in turbidity (**b**) before and (**a**) after nano treatment of sewing threads [25].

Cationic polymers have proven to have antiviral properties. Organic materials, such as chitosan, operate in a two-step mechanism by which the cationic chitosan binds with sialic acid in phospholipids, which restrains the movement of a microbial substance. Secondly, chitosan molecules penetrate the cell wall or coronae where they inhibit growth by preventing DNA-to-RNA transformation. In general, polymers tend to be inert; however, biodegradable polymers provide excellent eco-friendly alternatives with characteristics such as molecular penetrability, which make them ideal for disposable antiviral purposes. Chemical or natural oil extract-based systems typically operate by interfering with the viral cell wall or coronae, resulting in their destruction. These are typically encapsulated or could be directly applied as a finish. Through treatment with silver nanoparticles, even the sewing threads may be incorporated with anti-bacterial and anti-fungal properties. Figure 8 shows threads in Müeller-Hinton broth (a) after nano treatment and (b) before nano treatment [25].

Generally, it will be necessary to address the functionality of individual potential ways of preventing the spread of viruses in micro-atmosphere conditions under face masks (exhaled air contains about 6% water vapor and 4% CO_2_), which can be “controlled” by choosing the type of fibrous layer (e.g., moisture harvesting fibrous materials). Future active systems for efficient virus capture and destruction will be probably designed as a combination of different functional layers. This system will be used for the creation of medical protective clothing and only parts will be used for the construction of advanced RO face masks.

#### 2.3.5. Durability of the Face Masks

Durability or wear of materials used for the construction of face masks is one of the major factors deciding repeated use. It is not enough to test these materials according to standard laboratory procedures. It will be not possible to use only published information, because real textiles and plastics may contain several other auxiliary chemicals (as softeners, fillers, coupling agents, etc.) that change behavior during their use. It will be necessary to perform tests in simulated atmospheric conditions experienced by a face mask in use (approx. 37 °C, relative humidity exceeding 90% and external exposure to both sunlight and an artificial light source). These tests must be performed both on the materials before first use, during utilization (e.g., at half the number of their repeat use), and at end of life. This requires an accurate estimate of reuse cycles and a specification of possible sterilization methods. In addition to changes in material integrity and mechanical capture, it will be necessary to investigate possible solid, liquid, and gaseous particles released as a result of use and sterilization. It will be necessary to verify their presence in the human body after inhalation and to investigate their possible long-term accumulation.

#### 2.3.6. Effective Sterilization

An appropriate sterilization procedure must be included with any virus protection device. There are several options, but they require the specification of the required intensity of action, time, and possibly other data (concentration, wavelength, etc.). For example, washing information at a suitable temperature is not enough. Recommendations issued in Wuhan are sterilization by washing at 56 °C for at least 30 min (i.e., real washing at a longer time, including heating the bath). The problem is that these sterilization conditions were determined by research on a model virus [3]. It is known that the new coronavirus SARS-CoV-2 often behaves differently from the known coronavirus SARS-CoV-1, although it does not differ much in composition [3]. Therefore, information from sources investigating another type of virus (i.e., before 2020) cannot be used without verification.

Another possibility is the use of UV radiation. Germicidal lamps operate at wavelengths of 200–220 nm, but are in the UV C area, which is dangerous for the human body. The bond energy of C-C is 3.61 eV and the corresponding wavelength is 343.71 nm and, for the C-N bond, is 389 nm. In reality, these wavelengths are not effective for destroying bacteria and viruses in times up to one hour. The quickest sterilization is probably to use microwaves, but there are some problems with the evenness of heating (creation of hot spots). Chemical sterilizations are not very effective for face masks. A simple way is to use alkali conditions (pH over 11) during washing or boiling obtained by using standard soaps. There is as well the possibility to use dry heat (hot air or hot materials) above 100 °C.

**Figure 9:**
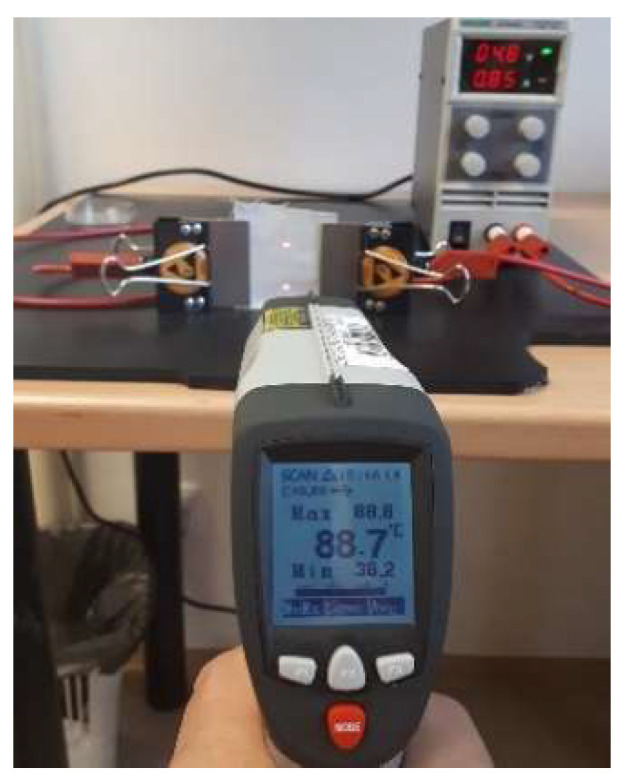
Generation of ohmic heat in a hybrid fabric.

One of the simplest ways is to use so-called ohmic heating of some conductive materials used for the construction of face masks textile layers. One good example of conductive materials is hybrid yarns containing fine metal fibers. In [26], hybrid yarns composed of conventional polyester fiber (59 wt %), cotton fiber (31 wt %), and BEKINOX stainless steel fibers (10 wt %) were used for the preparation of twill 2/1 weave hybrid fabric (warp sett 39 1/cm, weft sett 22 1/cm) with a planar mass 190 g m^−2^. The presence of the conductive component did not have a negative influence on comfort-related properties. This fabric was used for the generation of ohmic heat using two clamps, a power source, and a pyrometer (see Figure 9 for the characteristics of the hybrid fabric).

At 4.08 watts (4.8 volts and 0.850 amperes) the fabric sample with a dimension of 4 × 5 cm was heated to 88.7 °C and this temperature was maintained until switching off the power source. It is, therefore, possible to use textile fabric for filtration purposes and simultaneous realization of efficient generation of dry heat used for sterilization of face masks after use. Methods of effective sterilization must generally be in accordance with the materials used, regarding the formation of harmful products, in the event of possible degradation.

## 3. Recommendations

In times of absolute emergency, any process for preparing protective face masks is beneficial. However, without time constraints, it is necessary to look for comprehensive and proven solutions, based on systematic research and scientific data. Here, it is necessary to see the irreplaceable role of cross-disciplinary cooperation between academia and industry. In the current pandemic situation, many chronic problems can be easily triggered by the long-term use of unproven materials.

Several tests simulating the behavior of virus protection devices will need to be created or modified. Wearing comfort, related to breathability and humidification of face masks during wearing, should be considered as well. Solutions will require multi-disciplinary cooperation of textile and material scientists with toxicologists, biologists, and medical experts. A few other challenges will need to be addressed before protective face masks can be marketed that will do more good than harm. Regardless of whether protective face masks with longer-term re-use will be classified as medical materials, the responsible public authorities should require tests of both real functionality and safety for the recommended period of use. This is also a challenge for researchers and scientists who want to make a real contribution to ensuring the health of human society. We have used research data from the Technical University of Liberec as evidence to strengthen recommendations that can potentially save hundreds of thousands of lives, not just during the current COVID-19 pandemic, but also during similar situations in the future.

We hope that it will be possible to set up interdisciplinary teams across the scientific and research community, which will be able to comprehensively test the issue of protective face masks and provide consulting services to others, especially manufacturers. We should follow the time-tested but slightly modified rule for these exceptionally challenging times: Test 81 times but cut once.

## Figures and Tables

**Table 1:** Characteristics of typical membranes.

Characteristic	Micro Membrane	Nano Membrane
Fiber radius r [nm]	1520	140
Membrane thickness h [mm]	0.53	0.00185
Planar mass gsm [g/m^2^]	100	1.3
Volume porosity P_O_ [–]	0.83	0.36
S_r_ [m^2^/g]	1.196	12.987
S_sr_ [m^2^/m^2^]	119.62	16.88

**Table 2:** Planar density of prepared samples.

Type of Layer	Planar Density [g/m^2^]
1	76.9
2	37.1
3	26.2
4	20.2
5	15.7
6	13
7	11, 4

**Table 3:** Parameters of testing.

Parameter	Value	Unit
Filtration area	100	cm^2^
Filtration velocity	5.0	cm/s
Dust/aerosol	DEHS	-
Duration of test	60	S
Air flow	30.0	l/min
Range of particle size	0.12–3.5	Μm
Number of measurements	3	-
Temperature	21	°C
Humidity	54	%
Atmospheric pressure	1010	mbar

**Table 4:** Filtration efficiency characteristics.

Δp_0_ (Pa)	Pressure drop—the beginning of the test
Δp_1_ (Pa)	Pressure drop—end of the test (after 1 min)
E_mpps_ (%)	Filtration efficiency of particles with the highest penetration (minimum efficiency). Usually, these are particles with a size of 0.1–0.3 µm.
E_0.4_ (%)	Filtration efficiency of particles with a size of 0.4 µm (suitable for EN 779)
e_PM(1)_ (%)	Filtration efficiency of particles with the size of 0.3–1 µm in accordance with ISO 16890.
e_PM(2.5)_ (%)	Filtration efficiency of particles with the size of 0.3–2.6 µm in accordance with ISO 16890.

**Table 5:** Results of modified melt-blown filtration efficiency.

Sample	Type: 1	Type: 2	Type: 3	Type: 4	Type: 5	Type: 6	Type: 7
Δp_0_ (Pa)	53.00	33.00	22.00	17.00	14.00	11,00	10.00
Δp_1_ (Pa)	55.00	35.00	23.00	18.00	14.00	11.00	10.00
E_mpps_ (%)	60.85	40.18	29.03	21.92	17,55	14.79	15.57
E_0.4_ (%)	70.28	45.99	38.68	27.56	20,57	22.09	18.56
e_PM(1)_ (%)	74.92	53.46	44.39	34.92	24,46	22.96	22.32
e_PM(2.5)_ (%)	78.50	61.07	50.52	44.92	35,17	32.87	27.05

**Table 6:** Classification according to ISO 16890 standard.

Group	Size Range	Requirement	Examples
ISO e_PM1_	0.3 ≤ x ≤ 1	Minimum efficiency ≥ 50%	viruses, nanoparticles, exhaust gasses
ISO e_PM2.5_	0.3 ≤ x ≤ 2.5	Minimum efficiency ≥ 50%	bacteria, fungal and mold spores, pollen
ISO e_PM10_	0.3 ≤ x ≤ 10	Average efficiency ≥ 50%	pollen, fine sand
ISO _Coarse_	0.3 ≤ x ≤ 10	Average efficiency < 50%	sand, insect
